# Supplementary material for: Hepatocytes differentiate into intestinal epithelial cells through a hybrid epithelial/mesenchymal cell state in culture
Source: Nat Commun. 2024 May 15;15:3940. doi: 10.1038/s41467-024-47869-2 (PMC11096382; doi:10.1038/s41467-024-47869-2)
Supplement: Supplementary file 1 — Supplementary Information [file 41467_2024_47869_MOESM1_ESM.pdf]

SUPPLEMENTARY INFORMATION

Hepatocytes differentiate into intestinal epithelial cells through a hybrid epithelial/mesenchymal cell state in culture

Miura et al.

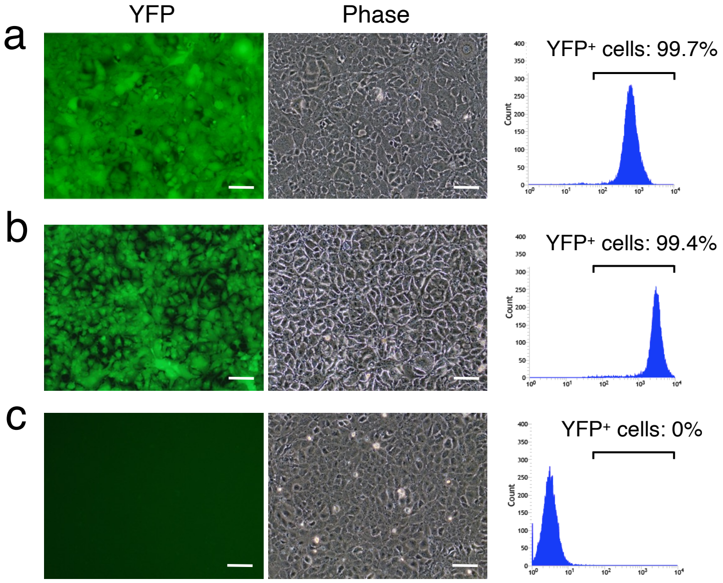

Supplementary Figure 1. Propagation of the progenies of adult mouse hepatocytes. (**a**-**c**) Representative fluorescence micrographs and morphologies of cells derived from hepatocytes isolated from two different TM-treated *Alb-CreER^{T2};R26R$^{YFP/+}$* mice (**a** and **b**) and from a wild-type mouse (**c**). Flow cytometry showing that almost all cells propagating in culture were derived from hepatocytes. Scale bars, 100 µm.

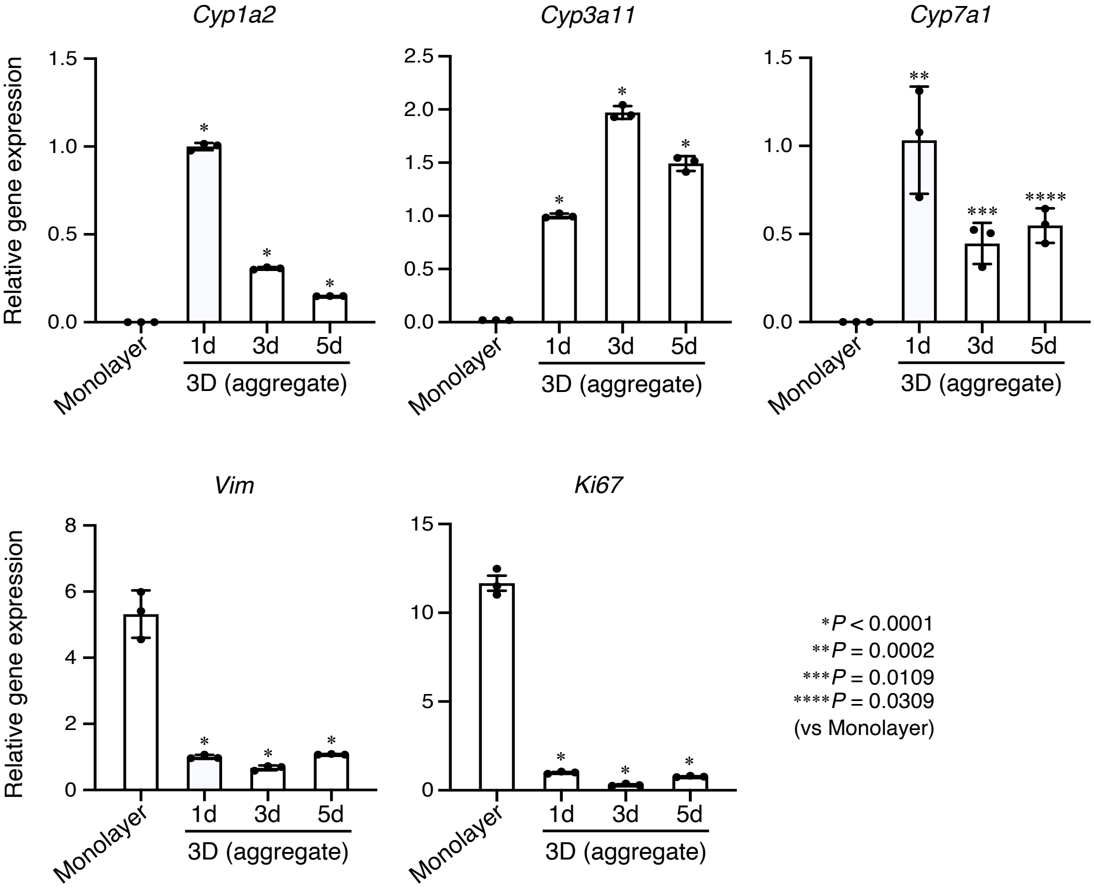

Supplementary Figure 2. Rapid induction of re-differentiation from dediHeps into hepatocytes in 3D culture. qPCR analyses of expression of *Cyp1a2*, *Cyp3a11*, *Cyp7a1*, *Vim*, and *Ki67* in dediHeps maintained in monolayer culture and dediHep aggregates 1 day (d), 3 d, and 5 d after initiation of 3D culture. All data were normalized to the values for dediHep aggregates (1 d) and are depicted as fold-changes. Data represent means ± SEM (n = 3 independent experiments). Statistical difference was determined by one-way analysis of variance followed by Dunnett's multiple comparison test. Source data are provided as a Source Data file.

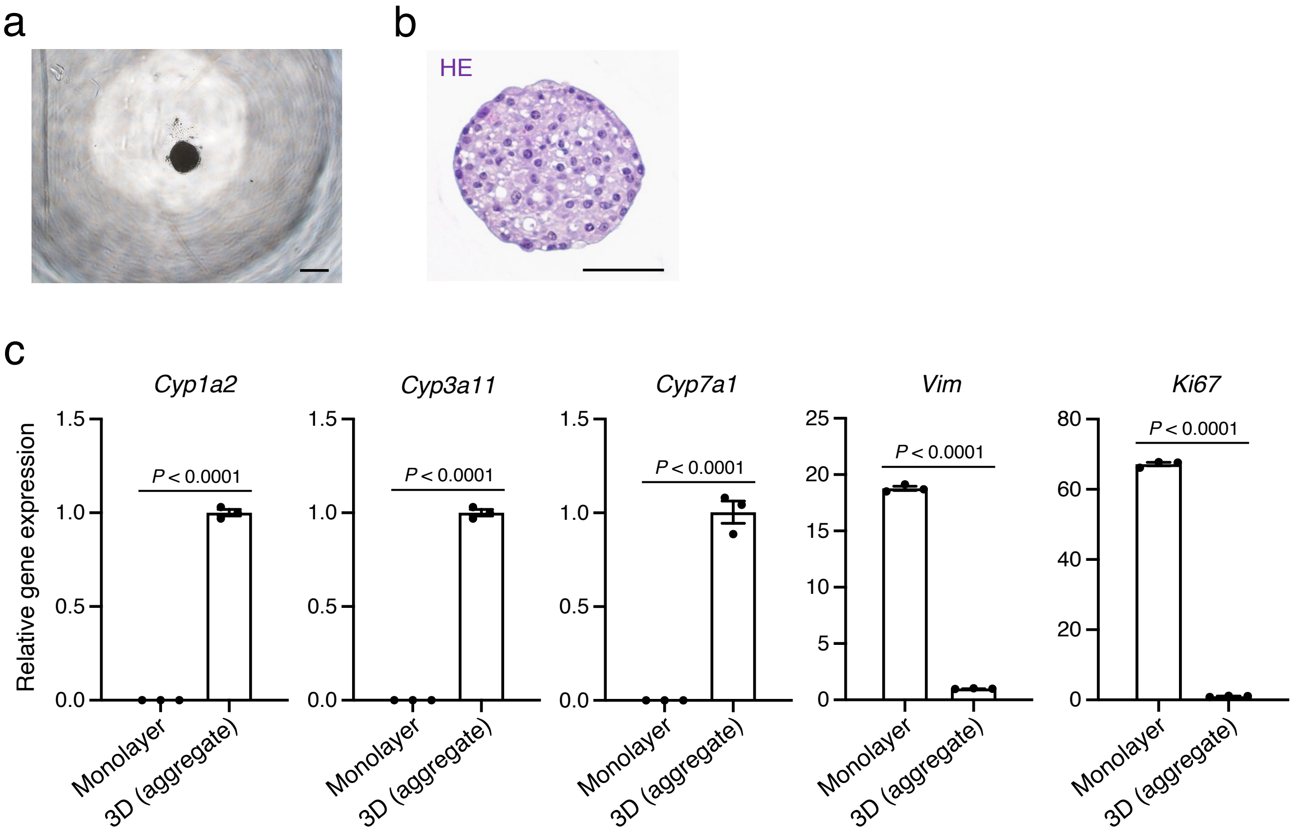

Supplementary Figure 3. Hepatocyte differentiation from dediHeps propagated in long-term monolayer culture. (**a**-**c**) dediHeps that have undergone 20 passages in long-term monolayer culture were used for cell-aggregate formation in 5-day 3D cultures. Representative morphology of a dediHep aggregate (**a**), a representative image of hematoxylin and eosin (HE)-stained dediHep aggregate (**b**), and qPCR analyses of expression of *Cyp1a2*, *Cyp3a11*, *Cyp7a1*, *Vim*, and *Ki67* in dediHep monolayer cultures and dediHep aggregates (**c**). All data were normalized to the values for dediHep aggregates and are depicted as fold-changes. Data represent means ± SEM (*n* = 3 independent experiments). Statistical difference was determined by two-sided Student's *t* test. Scale bars, 200 µm (**a**) and 50 µm (**b**). Source data are provided as a Source Data file.

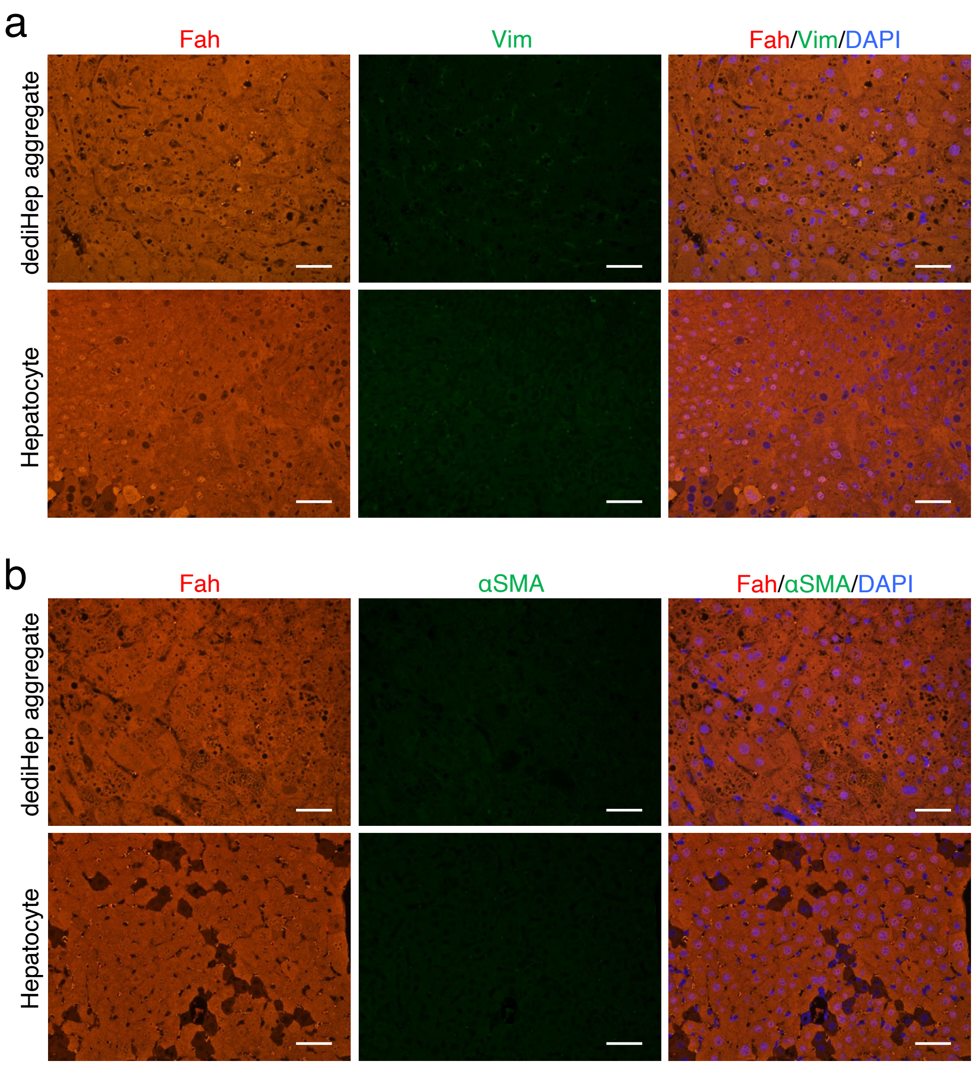

Supplementary Figure 4. dediHep-derived hepatocytes do not express mesenchymal cell markers *in vivo*. (**a** and **b**) Co-immunofluorescence staining of Fah with Vim (**a**) or αSMA (**b**) on liver sections of *Fah*$^{-/-}$ mice 3 months after transplantation of cells dissociated from dediHep aggregates or hepatocytes freshly isolated from adult mouse livers. DNA was stained with DAPI. Scale bars, 50 μm.

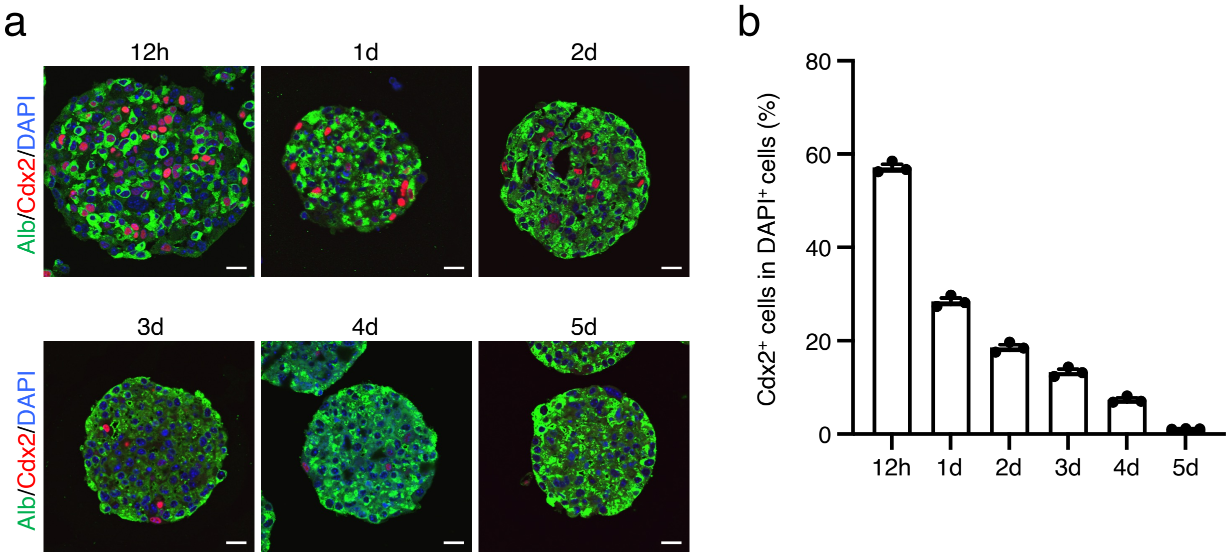

Supplementary Figure 5. Cdx2 gradually disappears from cells composing dediHep aggregates in 3D culture. (**a** and **b**) Co-immunofluorescence staining (Alb with Cdx2) of dediHep aggregates (**a**) and percentages of $Cdx2^{+}$ cells in these aggregates (**b**) 12 hours (h), 1 day (d), 2 d, 3 d, 4 d, and 5 d after initiation of 3D culture. DNA was stained with DAPI. Scale bars, 20 µm. Data represent means ± SEM (*n* = 3 independent experiments). Source data are provided as a Source Data file.

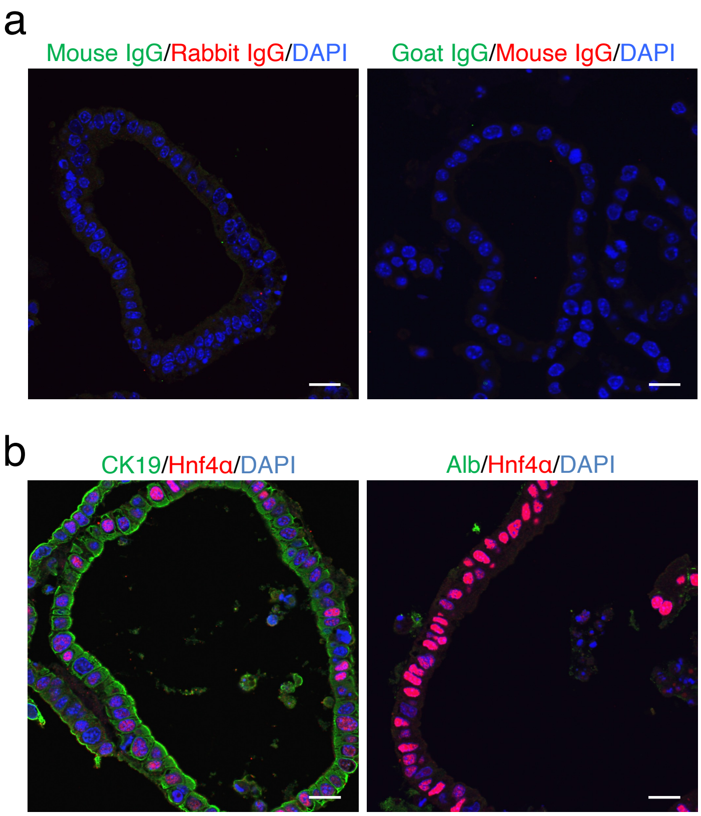

Supplementary Figure 6. dediHep-derived SOs are not biliary organoids. (**a**) As negative controls, we conducted co-immunofluorescence staining of dediHep-derived SOs using IgG isotype control antibodies. (**b**) Co-immunofluorescence staining (Hnf4a with CK19 or Alb) of dediHep-derived SOs. DNA was stained with DAPI. Scale bars, 20 μm.

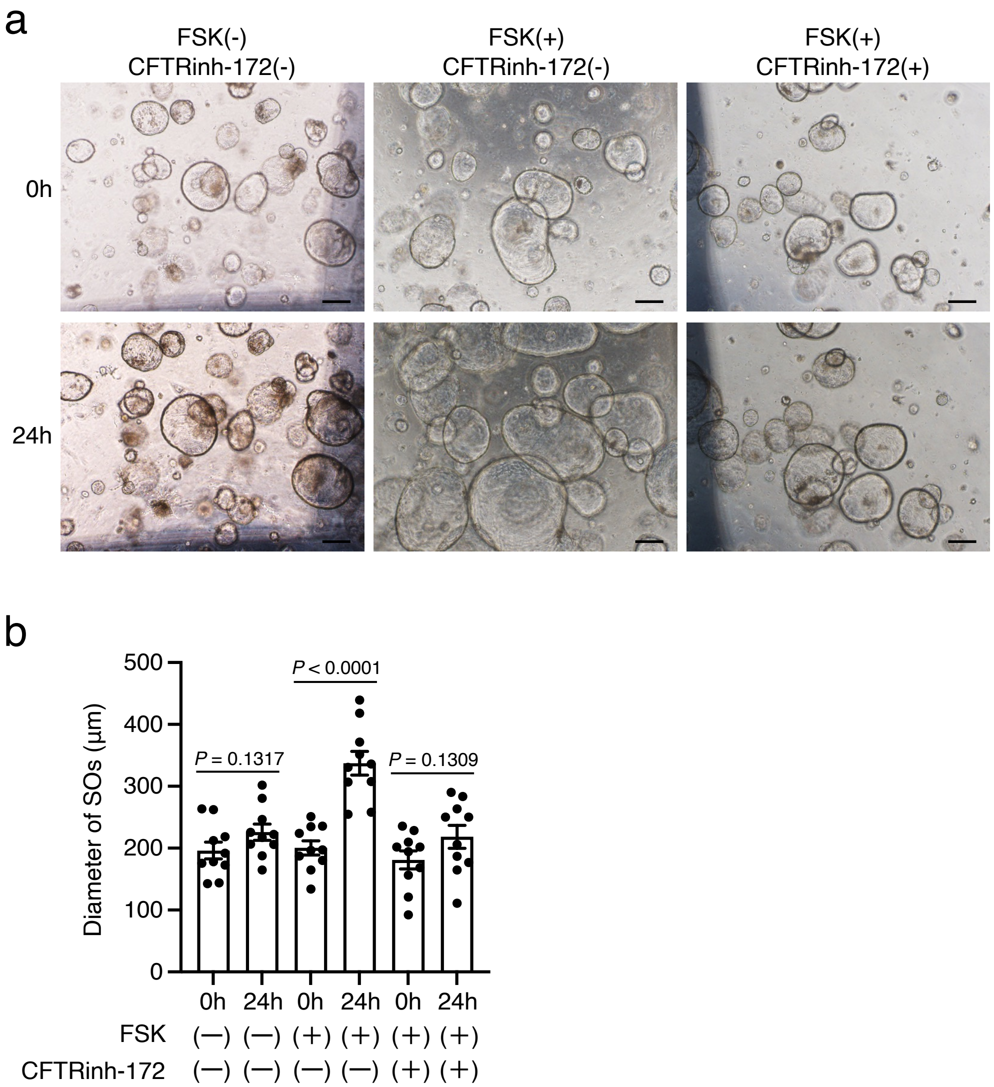

Supplementary Figure 7. dediHep-derived SOs are composed of functional intestinal epithelial cells. (**a**) Representative morphologies of dediHep-derived SOs before and after culture with or without FSK and/or CFTRinh-172 for 24 h. Scale bars, 200 µm. (**b**) The diameter of 10 SOs in each assay point of the indicated culture conditions was measured. Statistical difference was determined by two-sided Student's *t* test. Source data are provided as a Source Data file.

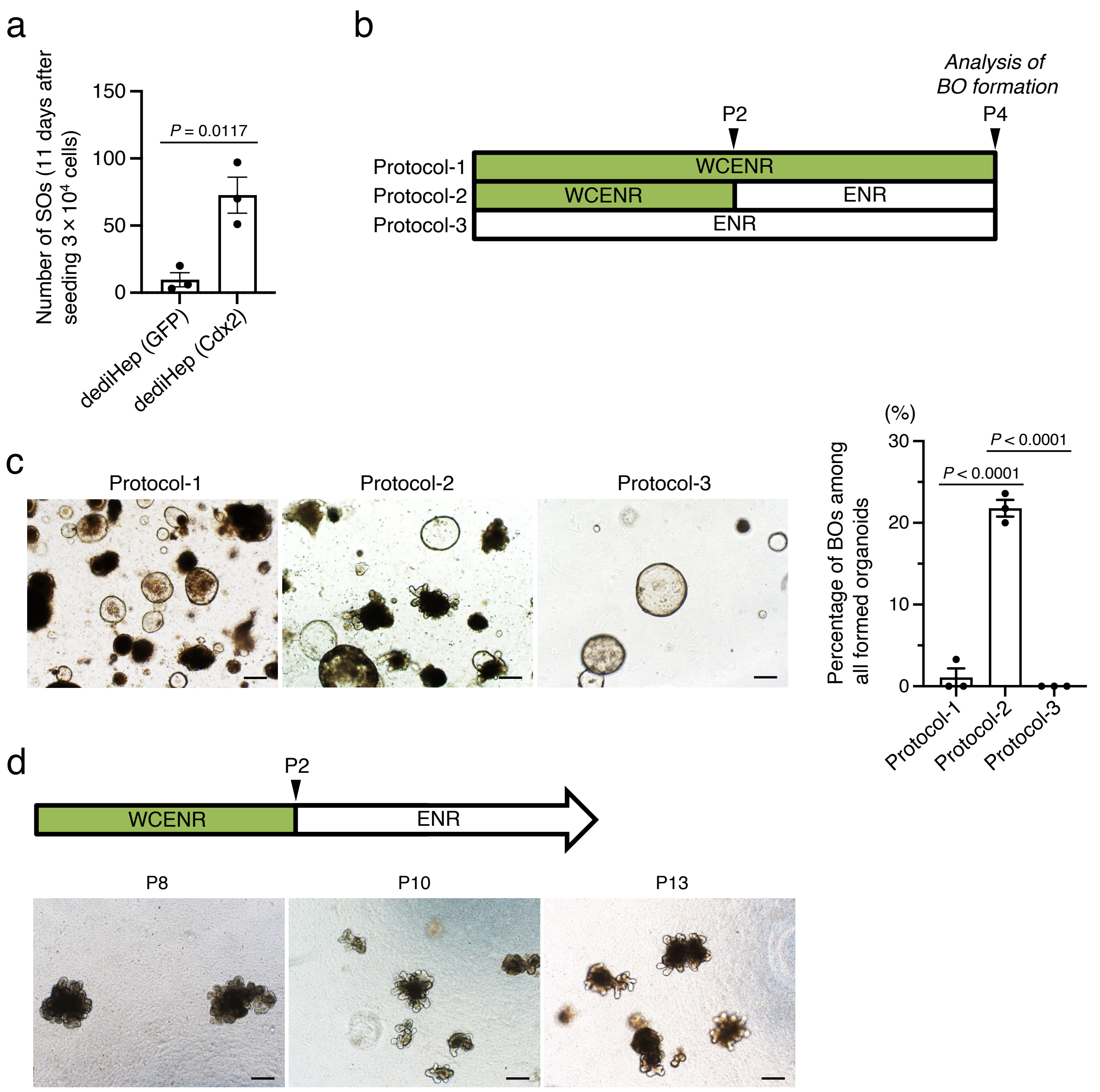

Supplementary Figure 8. Forced *Cdx2* expression in dediHeps promotes SO formation and allows BO formation by culturing with WCENR and subsequently excluding Wnt3a and CHIR99021 from the culture medium. (**a**) The number of SOs formed by dediHeps transduced with *GFP* or *Cdx2* (with *GFP*) at 11 days (d) after initiation of 3D culture with WCENR. (**b**) Schematic diagram of the experimental procedure. dediHeps transduced with *Cdx2* (with *GFP*) were embedded in Matrigel and cultured under the indicated conditions. At passage 4 (P4), the efficiency of formation of dediHep-derived BOs was analyzed. (**c**) Representative morphologies of dediHep-derived SOs and BOs at P4 after initiation of 3D culture. Graph at right depicts percentages of dediHep-derived BOs among all formed organoids. (**d**) Representative morphologies of dediHep-derived BOs at P8, P10, and P13 in long-term 3D culture. dediHeps transduced with *Cdx2* (with *GFP*) were cultured with ENR from P2. Scale bars, 200 µm. Data represent means ± SEM (n = 3 independent experiments). Statistical difference was determined by two-sided Student's t test (**a**) or one-way analysis of variance followed by Dunnett's multiple comparison test (**c**). Source data are provided as a Source Data file.

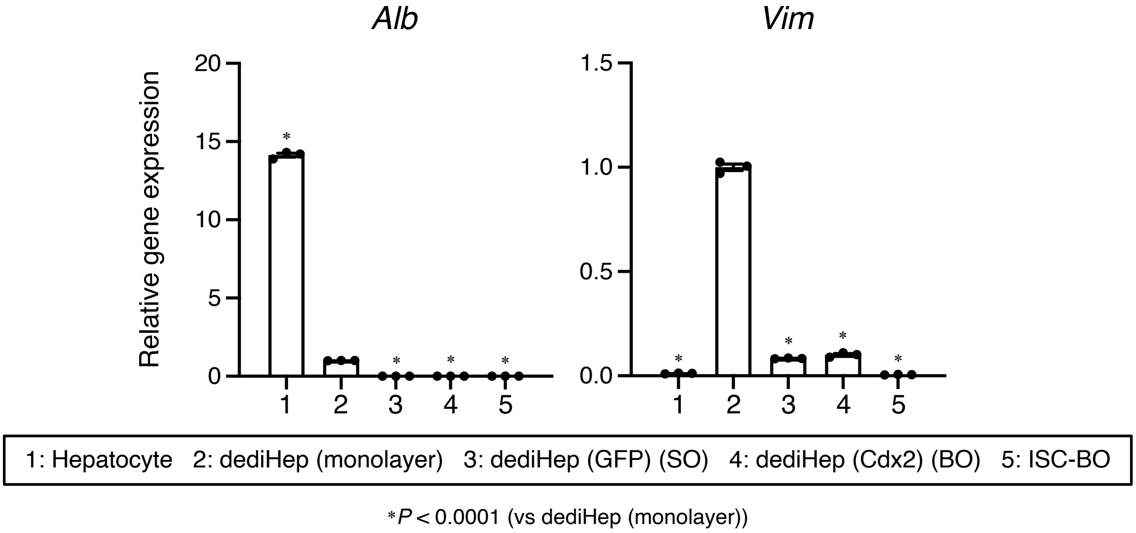

Supplementary Figure 9. Significant decrease in the expression of dediHep markers in BOs derived from exogenous *Cdx2*-expressing dediHeps. qPCR analyses of *Alb* and *Vim* expression in hepatocytes freshly isolated from adult mouse livers, dediHeps maintained in monolayer culture, SOs formed by dediHeps transduced with *GFP*, BOs formed by dediHeps transduced with *Cdx2* (with *GFP*), and ISC-derived BOs. All data were normalized to the values for dediHeps in monolayer culture and are depicted as fold-changes. Data represent means ± SEM (n = 3 independent experiments). Statistical difference was determined by one-way analysis of variance followed by Dunnett's multiple comparison test. Source data are provided as a Source Data file.

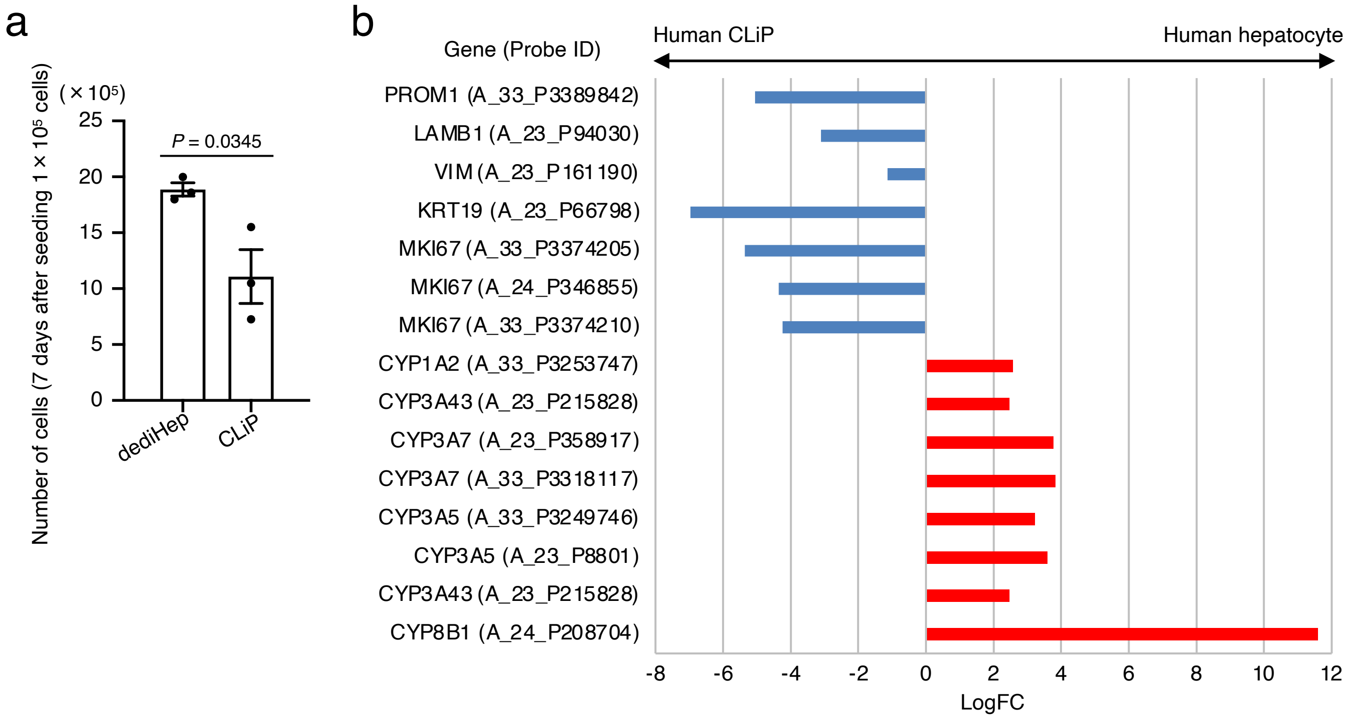

Supplementary Figure 10. The properties of cell proliferation and gene expression in CLiPs. (**a**) The number of mouse dediHeps and CLiPs at 7 days after plating. Data represent means ± SEM (n = 3 independent experiments). Statistical difference was determined by two-sided Student's t test. Source data are provided as a Source Data file. (**b**) Representative genes expressed differentially between human hepatocytes and CLiPs. Blue and red bars represent up- and down-regulated genes, respectively, in CLiPs.

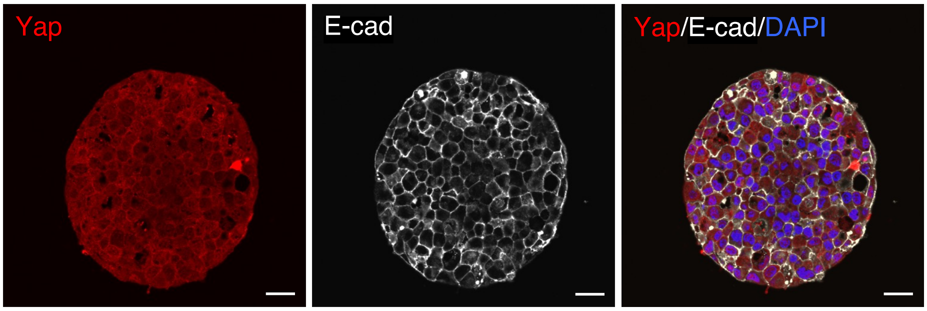

Supplementary Figure 11. Yap disappears from the nucleus of cells composing dediHep aggregates. Co-immunofluorescence staining (Yap with E-cad) of dediHep aggregates 5 days after initiation of 3D culture. DNA was stained with DAPI. Scale bars, 20 µm.

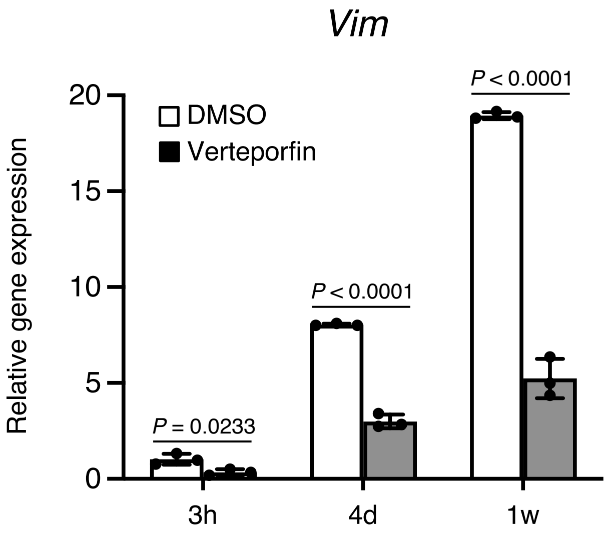

Supplementary Figure 12. Inhibition of Yap activity in hepatocyte cultures suppresses *Vim* expression. qPCR analysis of *Vim* expression in hepatocytes treated with DMSO or verteporfin (0.3 µM) for 3 hours (h), 4 days (d), and 1 week (w) after plating. All data were normalized to the values for hepatocytes cultured with DMSO for 3h and are depicted as fold-changes. Data represent means ± SEM (n = 3 independent experiments). Statistical difference was determined by two-sided Student's t test. Source data are provided as a Source Data file.

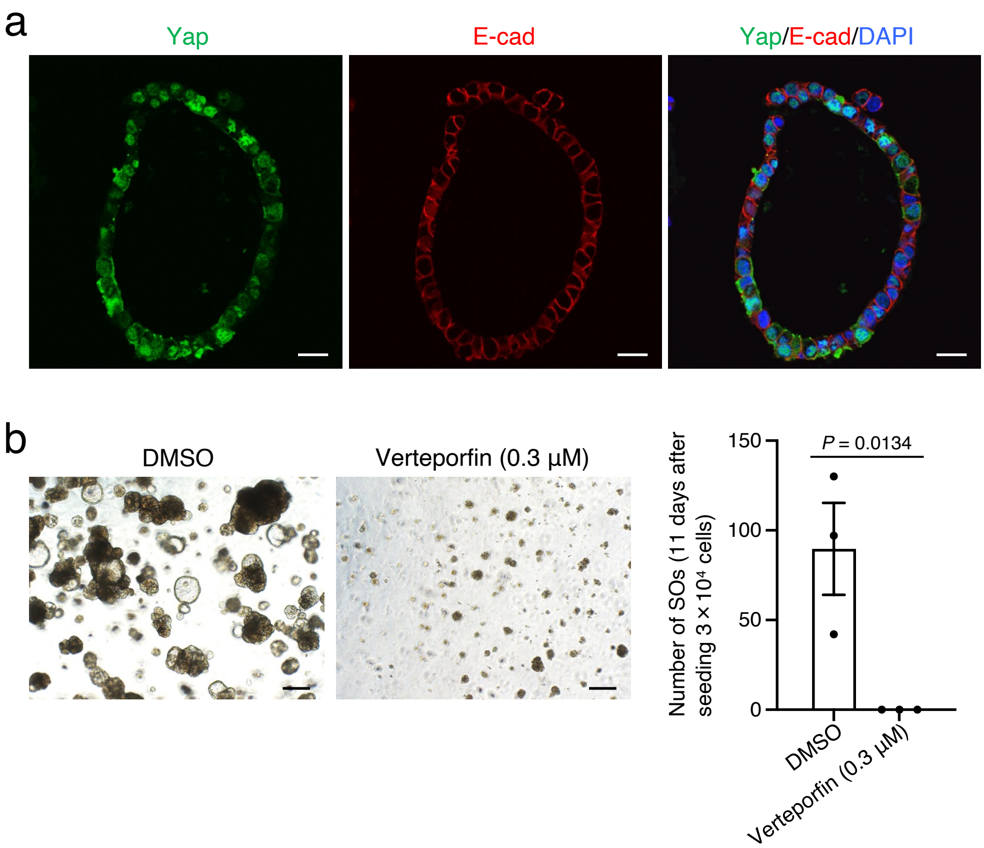

Supplementary Figure 13. Yap activation is required for the formation of dediHep-derived SOs. (**a**) Co-immunofluorescence staining (Yap with E-cad) of dediHep-derived SOs. DNA was stained with DAPI. (**b**) Representative morphologies and the number (right graph) of dediHep-derived SOs at 11 days after initiation of 3D culture with DMSO or verteporfin (0.3 µM). Data represent means ± SEM (*n* = 3 independent experiments). Statistical difference was determined by two-sided Student's *t* test. Scale bars, 20 µm (**a**) and 200 µm (**b**). Source data are provided as a Source Data file.

Supplementary Table 1. The list of antibodies.

	Antibody	Company	Catalog number	Dilution
**Primary antibodies**	Goat anti-Alb	Bethyl	A90-134	1:2000
	Rabbit anti-Alb	Biogenesis	0220-1829	1:2000
	Mouse anti-Hnf4α	PPMX	PP-H1415-00	1:1000
	Rabbit anti-CK19	[1]		1:2000
	Goat anti-GFP	NOVUS Biologicals	NB100-1770	1:4000
	Rabbit anti-E-cad	Cell Signaling Technology	3195S	1:500
	Mouse anti-E-cad	BD Biosciences	610182	1:1000
	Mouse anti-Vim	Sigma-Aldrich	V5255	1:1000
	Rabbit anti-Fah	Abcam	ab151998	1:2000
	Mouse anti-Cdx2	MBL	MU392AUC	1:100
	Rabbit anti-Sox9	Millipore	AB5535	1:1000
	Goat anti-Villin	Santa Cruz Biotechnology	SC-7672	1:1000
	Rabbit anti-Ki67	Abcam	ab15580	1:500
	Rabbit anti-Muc2	Santa Cruz Biotechnology	SC-15334	1:1000
	Rabbit anti-Klf5	GeneTex	GTX103289	1:100
	Goat anti-EphB2	R&D Systems	AF467	1:500
	Rabbit anti-Lyz	Dako	A0099	1:1000
	Goat anti-ChgA	Santa Cruz Biotechnology	SC-1488	1:500
	Rabbit anti-CC3	Cell Signaling Technology	9661	1:500
	Rabbit anti-YAP	Cell Signaling Technology	14074	1:500
	Rabbit anti-pHH3	Millipore	06-570	1:500
	Rabbit anti-GFP	MBL	598	1:500
	Normal mouse IgG	Santa Cruz Biotechnology	sc-2025	1:100 or 1:1000
	Normal rabbit IgG	Santa Cruz Biotechnology	sc-2027	1:500 or 1:1000
	Normal goat IgG	Santa Cruz Biotechnology	sc-2028	1:1000
	Guinea pig anti-CK8/18	Progen	GP11	1:500
	Mouse anti-αSMA	Sigma-Aldrich	A2547	1:2000
**Secondary antibodies**	Alexa 488-conjugated donkey anti-rabbit IgG	Invitrogen	A21206	1:2000
	Alexa 488-conjugated donkey anti-goat IgG	Invitrogen	A11055	1:2000
	Alexa 488-conjugated donkey anti-mouse IgG	Invitrogen	A21202	1:2000
	Alexa 488-conjugated goat anti-mouse IgM	Invitrogen	A21042	1:2000
	Alexa 555-conjugated donkey anti-rabbit IgG	Invitrogen	A31572	1:2000
	Alexa 555-conjugated donkey anti-goat IgG	Invitrogen	A21432	1:2000
	Alexa 555-conjugated donkey anti-mouse IgG	Invitrogen	A31570	1:2000
	Alexa 555-conjugated goat anti-mouse IgM	Invitrogen	A21426	1:2000
	Alexa 555-conjugated goat anti-Guinea Pig IgG	Invitrogen	A21435	1:2000
	HRP-conjugated goat anti-rabbit IgG	Dako	P0448	1:2000

Supplementary Table 2. The list of qPCR primers.

	Gene	Forward 5'-3'	Reverse 5'-3'
**qPCR primers**	*Alb*	GCACCAAGTGTTGTACACTTCCTGA	GCACTTGGTAACATGCTCACTCACT
	Cyp1a2	AGGGACACCTCACCTCACTGAATGG	GTCGATGGCCGAGTTGTTAT
	Cyp3a11	CTTTCCTTCACCCTGCATTC	CCAGGTATTCCATCTCCATCA
	Cyp7a1	TACAGAGTGCTGGCCAAGAG	GCAGAGCCTCCTTGATGATG
	Vim	TGCACGATGAAGAGATCCAG	AGCCACGCTTTCATACTGCT
	Ki67	CAGAAGAAATGGAAGCCAAAA	TGCAGATGCATCAAACTTGG
	Ctgf	CTGCAGACTGGAGAAGCAGAG	GCTCAAACTTGACAGGCTTGG
	Cyr61	GAAGTGCGTCCTTGTGGACA	CAGGAGCCGCAGTATTTGG
	GAPDH	TGTGTCCGTCGTGGATCTGA	TTGCTGTTGAAGTCGCAGGAG

Supplementary Reference

1. Sekiya, S. & Suzuki, A. Intrahepatic cholangiocarcinoma can arise from Notch-mediated conversion of hepatocytes. *J. Clin. Invest.* **122**, 3914–3918 (2012).
