# Supplementary material for: Hepatocytes differentiate into intestinal epithelial cells through a hybrid epithelial/mesenchymal cell state in culture
Source: Nat Commun. 2024 May 15;15:3940. doi: 10.1038/s41467-024-47869-2 (PMC11096382; doi:10.1038/s41467-024-47869-2)
Supplement: Supplementary file 3 — Reporting Summary [file 41467_2024_47869_MOESM3_ESM.pdf]

nature portfolio

Corresponding author(s): Atsushi Suzuki

Last updated by author(s): Apr 2, 2024

Reporting Summary

Nature Portfolio wishes to improve the reproducibility of the work that we publish. This form provides structure for consistency and transparency in reporting. For further information on Nature Portfolio policies, see our Editorial Policies and the Editorial Policy Checklist.

Statistics

For all statistical analyses, confirm that the following items are present in the figure legend, table legend, main text, or Methods section.

n/a	Confirmed	
☐	☒	The exact sample size (*n*) for each experimental group/condition, given as a discrete number and unit of measurement
☐	☒	A statement on whether measurements were taken from distinct samples or whether the same sample was measured repeatedly
☐	☒	The statistical test(s) used AND whether they are one- or two-sided<br>*Only common tests should be described solely by name; describe more complex techniques in the Methods section.*
☐	☒	A description of all covariates tested
☐	☒	A description of any assumptions or corrections, such as tests of normality and adjustment for multiple comparisons
☐	☒	A full description of the statistical parameters including central tendency (e.g. means) or other basic estimates (e.g. regression coefficient) AND variation (e.g. standard deviation) or associated estimates of uncertainty (e.g. confidence intervals)
☐	☒	For null hypothesis testing, the test statistic (e.g. *F*, *t*, *r*) with confidence intervals, effect sizes, degrees of freedom and *P* value noted<br>*Give P values as exact values whenever suitable.*
☒	☐	For Bayesian analysis, information on the choice of priors and Markov chain Monte Carlo settings
☒	☐	For hierarchical and complex designs, identification of the appropriate level for tests and full reporting of outcomes
☒	☐	Estimates of effect sizes (e.g. Cohen's *d*, Pearson's *r*), indicating how they were calculated

Our web collection on statistics for biologists contains articles on many of the points above.

Software and code

Policy information about availability of computer code

Data collection	IX71 and IX73 fluorescence microscopes (Olympus); FLUOVIEW FV10i confocal microscope (Olympus); CFX ConnectTM Real-Time PCR Detection System (Bio-Rad); NovaSeq 6000 system (Illumina); FACS Jazz (BD Biosciences)
Data analysis	celseq2 pipeline (v0.5.3.3); edgeR program (v4.0.2) on R software (v4.1.2); ggplot2 program (v3.4.4) on R software; DAVID website (v2023q3); iDEP website (v0.96); ggVolcanoR website (v1.0); GSEAPreranked program in GSEA software (v4.1.0); Microsoft Excel software (v16.69.1); JMP Pro software (v17.0.0); BD FACS Software sorter software; GraphPad Prism (v10.1.0)

For manuscripts utilizing custom algorithms or software that are central to the research but not yet described in published literature, software must be made available to editors and reviewers. We strongly encourage code deposition in a community repository (e.g. GitHub). See the Nature Portfolio guidelines for submitting code & software for further information.

Data

Policy information about availability of data

All manuscripts must include a data availability statement. This statement should provide the following information, where applicable:

- Accession codes, unique identifiers, or web links for publicly available datasets
- A description of any restrictions on data availability
- For clinical datasets or third party data, please ensure that the statement adheres to our policy

All data sets were deposited in the GEO database under Accession Number GEO: GSE248554 [https://www.ncbi.nlm.nih.gov/geo/query/acc.cgi?acc=GSE248554].

The publicly available data sets (Accession Number GEO: GSE133797 [https://www.ncbi.nlm.nih.gov/geo/query/acc.cgi?acc=GSE133797]) were also used in this study.

Human research participants

Policy information about studies involving human research participants and Sex and Gender in Research.

Reporting on sex and gender	NA
Population characteristics	NA
Recruitment	NA
Ethics oversight	NA

Note that full information on the approval of the study protocol must also be provided in the manuscript.

Field-specific reporting

Please select the one below that is the best fit for your research. If you are not sure, read the appropriate sections before making your selection.

- [x] Life sciences
- [ ] Behavioural & social sciences
- [ ] Ecological, evolutionary & environmental sciences

For a reference copy of the document with all sections, see nature.com/documents/nr-reporting-summary-flat.pdf

Life sciences study design

All studies must disclose on these points even when the disclosure is negative.

Sample size	The sample size of each experiment and statistical analysis have been shown in the figure legends and Methods section, and these sample sizes was ensured to detect differences between experimental groups.
Data exclusions	Genes expressed at very low levels (less than 0.5 counts-per-million in all samples) were removed from the data of CEL-seq2 before normalization with iDEP program.
Replication	All experiments were performed at least three times independently, except for the experiment performed twice to obtain the data shown in Figure 8i, and all attempts at replication were successful.
Randomization	All samples (cells, mice, etc.) used in the experiments were randomly allocated into different experimental groups.
Blinding	Experimenters were blinded to group allocation for transcriptome analysis. All other experiments were conducted in a non-blinded manner, because the research design was complicated, the researchers were restricted, and blinding feasibility was poor.

Reporting for specific materials, systems and methods

We require information from authors about some types of materials, experimental systems and methods used in many studies. Here, indicate whether each material, system or method listed is relevant to your study. If you are not sure if a list item applies to your research, read the appropriate section before selecting a response.

Materials & experimental systems

n/a	Involved in the study	
[ ]	[x]	Antibodies
[ ]	[x]	Eukaryotic cell lines
[x]	[ ]	Palaeontology and archaeology
[ ]	[x]	Animals and other organisms
[x]	[ ]	Clinical data
[x]	[ ]	Dual use research of concern

Methods

n/a	Involved in the study	
[x]	[ ]	ChIP-seq
[ ]	[x]	Flow cytometry
[x]	[ ]	MRI-based neuroimaging

Antibodies

Antibodies used	Antibodies used in this study are listed in Supplementary Table 1. Primary antibodies: Goat anti-Alb (Bethyl, A90-134, 1:2000), Rabbit anti-Alb (Biogenesis, 0220-1829, 1:2000), Mouse anti-Hnf4α (PPMX, PP-H1415-00, 1:1000), Rabbit anti-Mouse CK19 (made in Suzuki lab, Kyushu University, Japan, 1:2000), Goat anti-GFP (NOVUS Biologicals, NB100-1770, 1:4000), Rabbit anti-E-cad (Cell Signaling

	Technology, 3195S, 1:500), Mouse anti-E-cad (BD Biosciences, 610182, 1:1000), Mouse anti-Vim (Sigma-Aldrich, V5255, 1:1000), Rabbit anti-Fah (Abcam, ab151998, 1:2000), Mouse anti-Cdx2 (MBL, MU392AUC, 1:100), Rabbit anti-Sox9 (Millipore, AB5535, 1:1000), Goat anti-Villin (Santa Cruz Biotechnology, SC-7672, 1:1000), Rabbit anti-Ki67 (Abcam, ab15580, 1:500), Rabbit anti-Muc2 (Santa Cruz Biotechnology, SC-15334, 1:1000), Rabbit anti-Klf5 (GeneTex, GTX103289, 1:100), Goat anti-EphB2 (R&D Systems, AF467, 1:500), Rabbit anti-Lyz (Dako, A0099, 1:1000), Goat anti-ChgA (Santa Cruz Biotechnology, SC-1488, 1:500), Rabbit anti-CC3 (Cell Signaling Technology, 9661, 1:500), Rabbit anti-YAP (Cell Signaling Technology, 14074, 1:500), Rabbit anti-pHH3 (Millipore, 06-570, 1:500), Rabbit anti-GFP (MBL, 598, 1:500), Normal mouse IgG (Santa Cruz Biotechnology, sc-2025, 1:100 or 1:1000), Normal rabbit IgG (Santa Cruz Biotechnology, sc-2027, 1:500 or 1:1000), Normal goat IgG (Santa Cruz Biotechnology, sc-2028, 1:1000), Guinea pig anti-CK8/18 (Progen, GP11, 1:500), and Mouse anti-αSMA (Sigma-Aldrich, A2547, 1:2000). Secondary antibodies: Alexa 488-conjugated donkey anti-rabbit IgG (Invitrogen, A21206, 1:2000), Alexa 488-conjugated donkey anti-goat IgG (Invitrogen, A11055, 1:2000), Alexa 488-conjugated donkey anti-mouse IgG (Invitrogen, A21202, 1:2000), Alexa 488-conjugated goat anti-mouse IgM (Invitrogen, A21042, 1:2000), Alexa 555-conjugated donkey anti-rabbit IgG (Invitrogen, A31572, 1:2000), Alexa 555-conjugated donkey anti-goat IgG (Invitrogen, A21432, 1:2000), Alexa 555-conjugated donkey anti-mouse IgG (Invitrogen, A31570, 1:2000), Alexa 555-conjugated goat anti-mouse IgM (Invitrogen, A21426, 1:2000), Alexa 555-conjugated goat anti-Guinea Pig IgG (Invitrogen, A21435, 1:2000), and HRP-conjugated goat anti-rabbit IgG (Dako, P0448, 1:2000).
Validation	All commercial antibodies were validated by the vendors and documented by corresponding data sheets, and CK19 antibody was validated in the study reported previously (Sekiya, S. & Suzuki, A. J. Clin. Invest. 122, 3914–3918, 2012).

Eukaryotic cell lines

Policy information about cell lines and Sex and Gender in Research

Cell line source(s)	Plat-E cells (a gift from Dr. Toshio Kitamura) and 293T cells (a gift from Dr. Hiroyuki Miyoshi).
Authentication	Cell line authentication was performed based on their morphology, growth condition, and specific properties.
Mycoplasma contamination	All the cell lines were tested negative for mycoplasma contamination.
Commonly misidentified lines (See ICLAC register)	No commonly misidentified cell lines were used in the study.

Animals and other research organisms

Policy information about studies involving animals; ARRIVE guidelines recommended for reporting animal research, and Sex and Gender in Research

Laboratory animals	C57BL/6 (Clea Japan, Tokyo, Japan), Alb-Cre (The Jackson Laboratory, Bar Harbor, ME), Alb-CreERT2 (a gift from Drs. Pierre Chambon and Daniel Metzger), R26RYFP/YFP (a gift from Dr. Frank Costantini), Fah-/- (RBRC05362) (RIKEN, Japan), NSG (NOD.Cg-PrkdcscidIl2rgtm1Wjl/SzJ) (Charles River Laboratories, Wilmington, MA), and Tgfbr2fl/fl (a gift from Dr. Jürgen Roes) mice were used in this study. Mice were housed in groups of 2-4 per cage in a 12 h light/dark cycle (08:00-20:00 light; 20:00-8:00 dark), with controlled room temperature (22 ± 4°C) and relative humidity (60%).
Wild animals	No wild animals were used in the study.
Reporting on sex	Female mice (8–12 weeks-old) were used as recipient mice for transplantation experiments, while male mice (8 and 10 weeks-old) were used for other experiments.
Field-collected samples	No field-collected samples were used in this study.
Ethics oversight	The experiments were approved by the Kyushu University Animal Experiment Committee, and the care of the animals was in accordance with institutional guidelines.

Note that full information on the approval of the study protocol must also be provided in the manuscript.

Flow Cytometry

Plots

Confirm that:

- [x] The axis labels state the marker and fluorochrome used (e.g. CD4-FITC).
- [x] The axis scales are clearly visible. Include numbers along axes only for bottom left plot of group (a 'group' is an analysis of identical markers).
- [x] All plots are contour plots with outliers or pseudocolor plots.
- [x] A numerical value for number of cells or percentage (with statistics) is provided.

Methodology

Sample preparation	Single cells collected from monolayer cultures were incubated with PI and washed with PBS.

Instrument	FACS Jazz (BD Biosciences)
Software	BD FACS Sortware sorter software
Cell population abundance	The purity of PI-negative and YFP-positive cells was confirmed by comparing with negative controls (PI-untreated, wild-type mouse-derived dediHeps).
Gating strategy	Gating strategy is shown in Supplementary Figure 1. Cells were initially gated on FSC/SSC to exclude debris, and then only single cells were gated. YFP-positive and negative cells were identified based on the data of negative controls (dediHeps derived from wild-type mice).

☒ Tick this box to confirm that a figure exemplifying the gating strategy is provided in the Supplementary Information.
